# The method of purifying bioengineered spider silk determines the silk sphere properties

**DOI:** 10.1038/srep28106

**Published:** 2016-06-17

**Authors:** Katarzyna Jastrzebska, Edyta Felcyn, Maciej Kozak, Miroslaw Szybowicz, Tomasz Buchwald, Zuzanna Pietralik, Teofil Jesionowski, Andrzej Mackiewicz, Hanna Dams-Kozlowska

**Affiliations:** 1 Chair of Medical Biotechnology, Poznan University of Medical Sciences, 61-688 Poznan, Poland; 2 NanoBioMedical Centre, Adam Mickiewicz University, 61-614 Poznan, Poland; 3 BioContract Sp. z o.o., 61-051 Poznan, Poland; 4 Department of Macromolecular Physics, Adam Mickiewicz University, 61-614 Poznan, Poland; 5 Joint Laboratory for SAXS Studies, Adam Mickiewicz University, 61–614 Poznan, Poland; 6 Faculty of Technical Physics, Poznan University of Technology, 60-965 Poznan, Poland; 7 Faculty of Chemical Technology, Institute of Chemical Technology and Engineering, Poznan University of Technology, 60-965 Poznan, Poland; 8 Department of Diagnostics and Cancer Immunology, Greater Poland Cancer Centre, 61-688 Poznan, Poland

## Abstract

Bioengineered spider silks are a biomaterial with great potential for applications in biomedicine. They are biocompatible,biodegradable and can self-assemble into films, hydrogels, scaffolds, fibers, capsules and spheres. A novel, tag-free, bioengineered spider silk named MS2(9x) was constructed. It is a 9-mer of the consensus motif derived from MaSp2–the spidroin of *Nephila clavipes* dragline silk. Thermal and acidic extraction methods were used to purify MS2(9x). Both purification protocols gave a similar quantity and quality of soluble silk; however, they differed in the secondary structure and zeta potential value. Spheres made of these purified variants differed with regard to critical features such as particle size, morphology, zeta potential and drug loading. Independent of the purification method, neither variant of the MS2(9x) spheres was cytotoxic, which confirmed that both methods can be used for biomedical applications. However, this study highlights the impact that the applied purification method has on the further biomaterial properties.

An ideal drug carrier should be made of a material that meets a number of requirements, such as biocompatibility, biodegradability, and well-defined chemical and physical properties. The production process of the carrier should be controllable, repeatable and cost-effective; most importantly, it should use mild, biocompatible reagents and omit those that may be retained in the carrier, which could cause toxic effects.

Spider silk protein seems to be an excellent candidate for many biomedical applications, including drug delivery carriers. Spider silk is known for its mechanical properties, such as high toughness, elasticity and mechanical strength. As a protein-based material, silk is biocompatible and biodegradable^1^. Although native spider silk is difficult to obtain in a pure form and in sufficient quantities, the development of recombinant spider silk production techniques and purification methods successfully solved the accessibility problem and paved the way for further research^2^,^3^,^4^.

A variety of heterologous expression hosts have been used to produce different silk variants^2^,^3^. Bioengineered silks produced in heterologous hosts (most commonly *Escherichia coli*) can form aggregates within the cell (inclusion bodies) and can be secreted in a soluble form to the cytoplasm or outside the cell to the culture media. For recovery of silks deposited in the cytoplasm, different purification methods were designed: affinity chromatography (the most common)^5^,^6^,^7^,^8^, thermal extraction^9^,^10^ and acidic extraction^10^,^11^. The first method requires an affinity tag, commonly His-tag, in the recombinant protein sequence and involves bacterial cell lysis and subsequent affinity chromatography on a column with Zn^2+^, Cu^2+^, Co^2+^ and Ni^2+^ metal ions. The process of purification occurs under native^6^,^8^ or denaturing^5^,^7^ conditions and is followed by dialysis. Despite the simplicity and robustness of this technique, the proteins obtained by this method possess an affinity domain that may alter the protein properties, disrupt their function or cause protein cytotoxicity. Indeed, the fibers made of bioengineered spider silk with His-tag were brittle and difficult to manipulate, whereas fibers without His-tag presented good mechanical properties^12^. Thus, an additional step of tag-removal may be needed.

Thermal and acidic extraction methods are based on innate silk properties: thermal stability and resistance to acid. High temperature and concentrated acid cause precipitation of expression host proteins, whereas silk proteins remain soluble^9^,^10^,^11^. These methods yield pure silk protein with high efficiency. However, as the greatest advantage, both methods circumvent the need for a tag sequence, which is ultimately favorable for biomedical applications.

In our previous study, we investigated the relationship between purification method and cytotoxicity of the MaSp1-based bioengineered silk proteins (15X and 6X)^10^. We showed that thermal and acidic extraction methods yield pure, non-toxic and non-immunogenic soluble silk variants when tested over a wide range of concentrations. At the highest concentration tested (1000 μg/ml), the proteins displayed some cytotoxicity, and acid extraction produced silks more toxic than these purified by the thermal method. However, independent of the purification method, all silk variants assembled into films that supported cell growth. Thus, both methods were considered to be suitable for obtaining silk proteins to produce safe biomaterials for biomedical applications^10^.

Although in nature silk proteins form only fibers, researchers developed several approaches for *in vitro* recombinant silk polymerization to produce various morphological forms such as fibers, films, hydrogels, sponges, scaffolds, capsules and spheres^13^,^14^. The silk spheres (particles) are suitable for drug delivery applications. Various approaches to produce silk spheres have been explored. Different silk variants have been used, including the following: (i) regenerated silkworm silk^15^,^16^,^17^; (ii) bioengineered spider silks such as eADF4(C16), which is based on the ADF4 protein of the European garden spider *Araneus diadematus*^18^,^19^,^20^,^21^; and (iii) MS1 silk variants adopted from the MaSp1 dragline spidroin of the Golden orb weaver - *Nephila clavipes*^22^. Various methods for sphere formation have been applied, including the following: (i) desolvation with an organic solvent^16^,^23^,^24^, (ii) sonication of silk/PVA blend film^25^; (iii) microfluidics^26^; (iv) lipid templates^27^, and (v) salting out with potassium phosphate ions in the presence of shear forces triggered by mixing^18^,^19^,^20^,^22^. Because the last method mimics the process of natural silk thread spinning, it provides the most biocompatible method for silk particle production. Using this method, it has been found that the protein concentration, phosphate concentration and pH of potassium phosphate play a role in the particle formation process^15^,^19^. The amino acid sequence of the recombinant silk also seems to be a critical determinant of the particle properties^28^. Yet, a great number of factors that can affect the sphere formation process and the spheres’ properties are not well understood.

In this study, we used thermal and acidic extraction methods to purify a novel, tag-free, bioengineered silk protein named MS2(9x)–a 9-mer of the consensus motif derived from MaSp2–the second spidroin of *N. clavipes* dragline silk. We tested the ability of the new bioengineered silk, MS2(9x), to form stable spherical particles. We investigated and highlighted the influence of the silk protein purification method on the sphere formation process, particle properties and drug loading potential. We indicated that the secondary structure of the soluble silk protein is a result of the protein purification method applied, which may determine the biomaterial’s properties. This finding may be of great importance for controlling biomaterial features.

## Results

### MS2(9x) silk gene construction, protein production and purification

**Figure 1:**
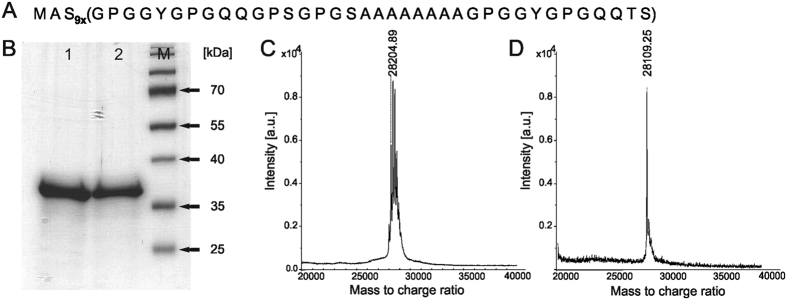
(**A**) Amino acidic sequence of the MS2(9x) protein; (**B**) 12.5% acrylamide SDS-PAGE gel analysis of MS2(9x) proteins; 1, MS2(9x) 80/20 - the protein purified with thermal method (80/20); 2, MS2(9x) PA, the protein purified with acidic method (PA); M, molecular weight marker (PageRuler, Fermentas); (**C**) MALDI-TOF spectrum of the MS2(9x) 80/20 protein; (**D**) MALDI-TOF spectrum of the MS2(9x) PA protein.

Figure 1A shows the amino acid sequences of the novel bioengineered silk MS2(9x). The protein was purified from the bacterial cells using two methods: (i) thermal extraction method, 80/20, for 80 °C denaturation temperature and 20% ammonium sulfate precipitation, and (ii) acidic extraction method, PA, Propionic Acid. The purified silk protein was accordingly named MS2(9x) 80/20 and MS2(9x) PA. An amount of silk obtained from 1 g of a bacterial pellet was 0.77 mg and 0.88 mg for the 80/20 and the PA method, respectively. The quality of purified silk was similar for both methods as neither degradation nor impurities were observed in the SDS-PAGE gel (Fig. 1B). Although the SDS-PAGE analysis indicated a higher than expected molecular weight of MS2(9x) silk, the MALDI-TOF results confirmed that the molecular weight of both–MS2(9x) 80/20 and MS2(9x) PA proteins was in agreement with the calculated value of 28.15 kDa (Fig. 1C,D).

### Secondary structure of the soluble spider silk proteins

**Figure 2:**
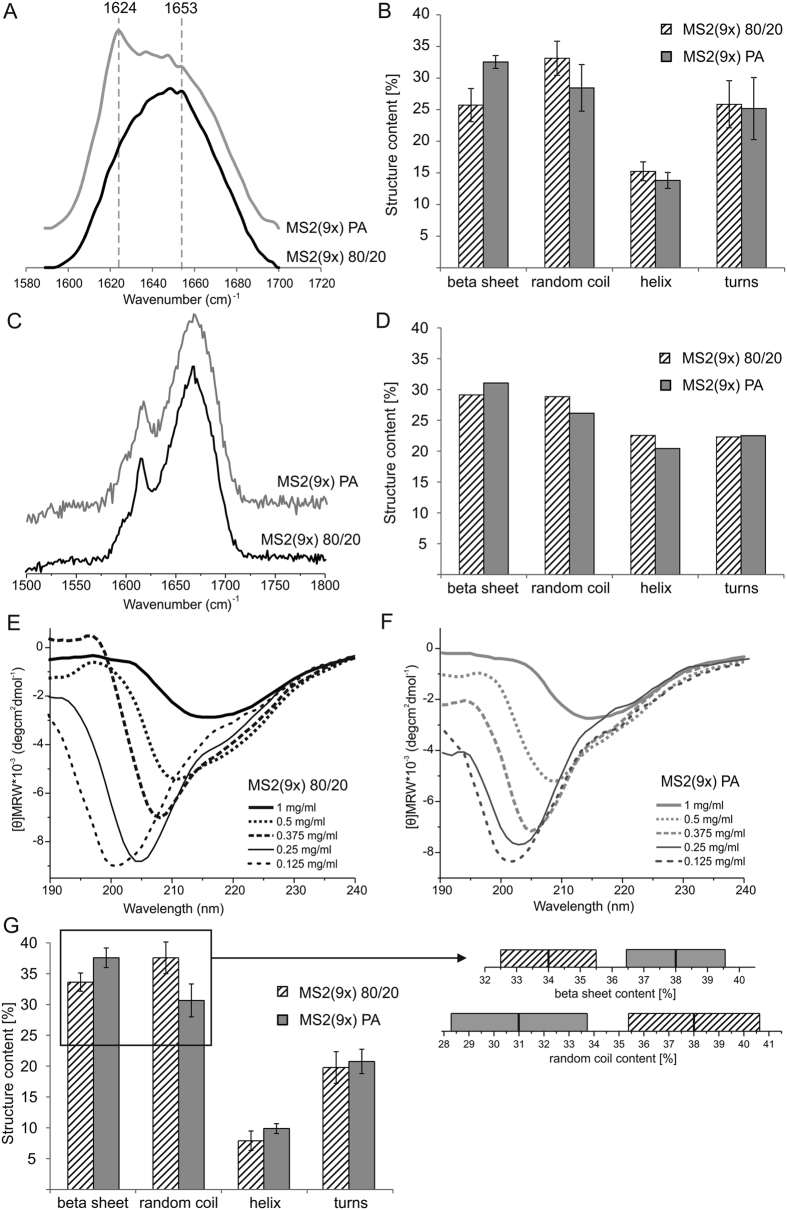
Secondary structure analysis of the soluble MS2(9x) 80/20 and MS2(9x) PA variants. (**A**) FTIR, (**C**) Raman and (**E**,**F**) CD spectra of the MS2(9x) 80/20 and MS2(9x) PA proteins. (**A**) Bands at 1624 and 1653 cm^−1^ indicate β-sheet and random coil, respectively. Proteins were measured at concentration of 5 mg/ml for FTIR and Raman analysis and for CD at the concentrations from 0.125 to 1 mg/ml. (**B**,**D**,**G**) The secondary structure composition of the MS2(9x) 80/20 and MS2(9x) PA proteins calculated from FTIR (**B**), Raman (**D**) and CD (**G**) spectra. (**G**) CD spectra of samples at concentration of 1 mg/ml were used for calculations of the secondary structure content. In the inset - the graphical version of the statistical hypothesis testing for average values and their standard deviation values are presented; for both fractions for which the changes were statistically significant (beta sheet, random coil). Bars indicate the standard deviations of the estimated content of secondary structure elements.

The secondary structure of the MS2(9x) proteins purified by thermal and acidic method was analyzed using Fourier Transform Infrared (FTIR), Raman and Circular Dichroism (CD) spectroscopy (Fig. 2). Every method showed higher β-sheet and lower random coil content for MS2(9x) PA protein comparing with MS2(9x) 80/20 protein, while content of helix and turns was similar for both analyzed proteins (Fig. 2B,D,G). The Supplementary Material shows the detailed information concerning circular dichroism measurement and data analysis (deconvolution of CD spectra and analysis of secondary structure).

Moreover, obtained circular dichroism spectra have revealed that the secondary structure of both variants of the MS2(9x) proteins was strongly dependent on protein concentration (Fig. 2E,F). The most common elements of secondary structures i.e. helixes and beta sheets gave rise to negative ellipticity in the spectral range of 210–220 nm, as shown at spectra of samples of the highest concentration (1 mg/ml). Dilution of both proteins caused a shift of this band toward lower wavelengths and an increased the intensity of this negative band, what indicated increasing contribution of random coil (Fig. 2E,F).

### Zeta potential (ZP) of the soluble spider silk proteins

**Figure 3:**
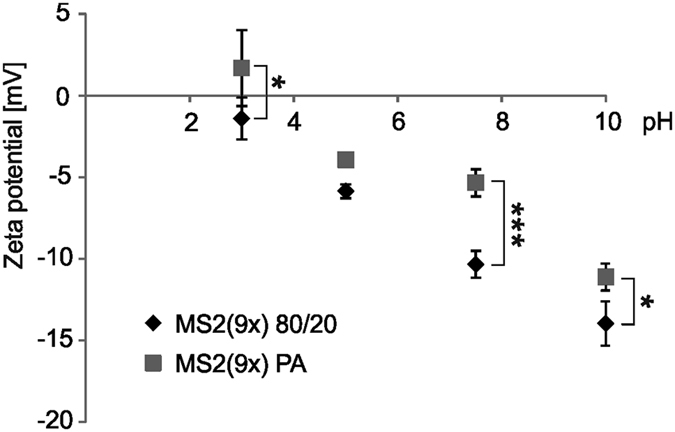
Zeta potential of the soluble variants of MS2(9x) proteins measured at various pH values. Proteins were measured three times in triplicate at 25 °C at the concentration of 2.5 mg/ml. *Indicates statistical significance with p < 0.05, and ***p < 0.001.

Soluble proteins MS2(9x) 80/20 and PA showed different ZP when measured at pH 3, 5, 7.5 and 10 (Fig. 3). Protein purified with thermal method had lower ZP at all analyzed pH values. The biggest difference between the ZP of both proteins was observed at pH of 7.5. and it was −10,3 mV and −5,3 mV for MS2(9x) 80/20 and MS2(9x) PA, respectively (Fig. 3). The differences were significant.

### Sphere formation and morphology

Upon addition of a potassium phosphate buffer and application of mixing conditions, both MS2(9x) 80/20 and MS2(9x) PA proteins formed spherical particles. The spheres remained stable after dialysis against water. We analyzed several different sphere processing conditions applying three variables: (1) a phosphate buffer concentration, (2) an initial silk concentration, and (3) a pH value of the phosphate buffer.

**Figure 4:**
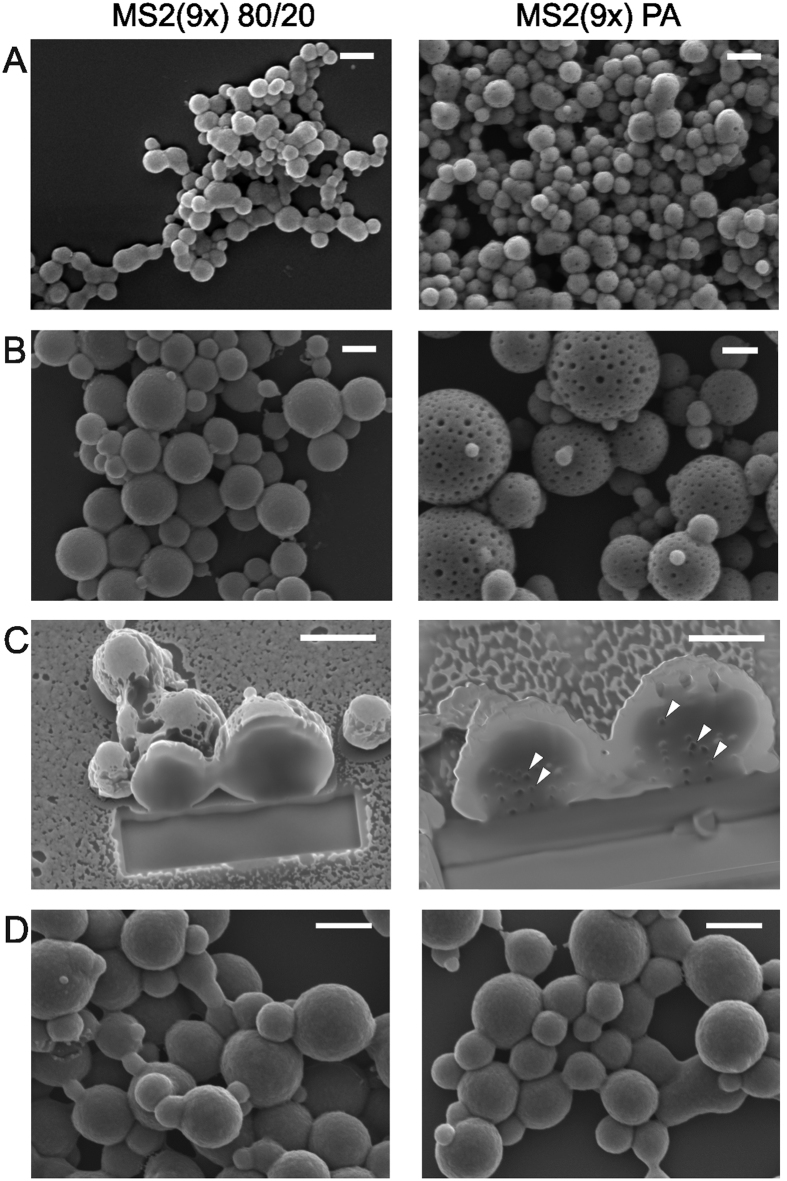
Morphology of the MS2(9x) 80/20 and MS2(9x) PA spheres under a scanning electron microscope. Spheres were formed by mixing of silk protein at an initial concentration of: (**A**) 0.5 mg/ml, and (**B**) 5 mg/ml with 2 M potassium phosphate at a pH of 8. (**C**) Spheres formed by mixing of silk proteins at an initial concentration of 5 mg/ml with 2 M potassium phosphate at a pH of 8 and cross-sectioned by a focused ion beam. White arrowheads show pores inside the MS2(9x) PA spheres. (**D**) Spheres made of silk proteins after additional processing steps. Soluble MS2(9x) proteins purified with 80/20 and PA methods were precipitated with 20% ammonium sulfate, resolubilized with 6 M guanidine thiocyanate and dialyzed against 10 mM Tris-HCl at a pH of 7.5. Spheres were formed at 2.5 mg/ml initial silk concentration and 2 M potassium phosphate at a pH of 8. Scale bars: 1 μm.

**Figure 5:**
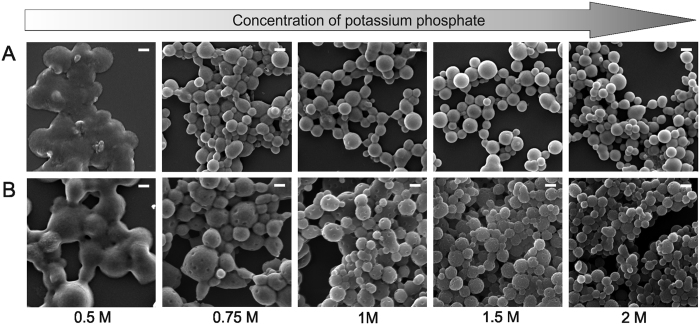
Scanning electron microscope images of the MS2(9x) bioengineered silk spheres formed from the MS2(9x) silk protein (at an initial concentration of 2.5 mg/ml) at various concentrations of potassium phosphate buffer with a pH of 8. (**A**) MS2(9x) 80/20 spheres and (**B**) MS2(9x) PA spheres Scale bars: 1 μm.

Independent of the initial silk concentration, the PA-purified MS2(9x) formed approximately 35% larger spheres than 80/20-purified silk (Figs 4 and 5). Moreover, MS2(9x) PA spheres presented a porous-like surface (particularly visible in the case of larger particles), whereas surfaces of the MS2(9x) 80/20 silk spheres were smooth (Fig. 4B). Similarly, after FIB milling of the particles, the cross-sections revealed pores inside the MS2(9x) PA-particles, whereas the MS2(9x) 80/20 spheres had a solid structure and no porosity was observed (Fig. 4C). Interestingly, the morphology of MS2(9x) PA spheres was modified after the additional processing step for the soluble silk proteins. Previously purified MS2(9x) 80/20 and MS2(9x) PA proteins were precipitated, re-solubilized with 6 M guanidine thiocyanate, and dialyzed against Tris buffer. Spheres formed from MS2(9x) 80/20 and MS2(9x) PA proteins that were subjected to the additional purification processing presented similar size and morphology (Fig. 4D). No porosity was observed on the surface of the MS2(9x) PA spheres.

**Figure 6:**
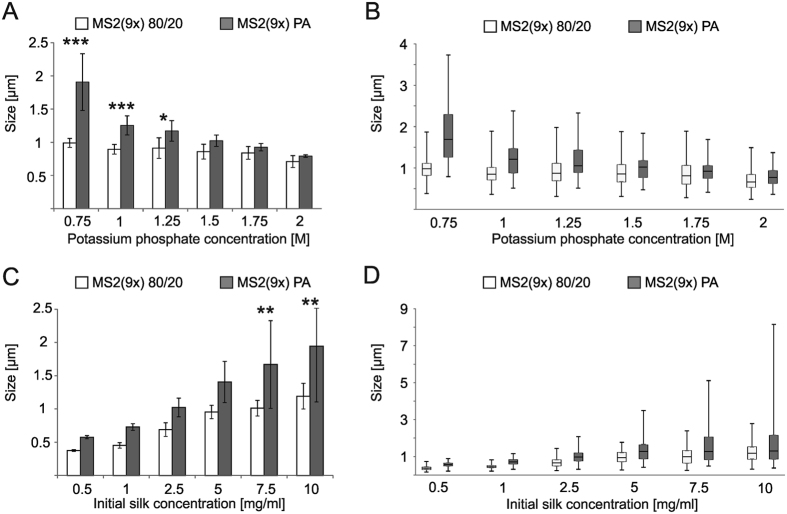
Size and size distribution of the MS2(9x) silk spheres. Spheres were prepared by mixing: (**A**,**B**) silk protein (at an initial concentration of 2.5 mg/ml) with various potassium phosphate concentrations at pH 8 and (**C**,**D**) silk protein at various concentrations with 2 M potassium phosphate at pH 8. (**A**,**C**) Size of spheres, error bars show the standard deviation from the mean diameter of the spheres from three independent experiments, ***indicates statistical significance with p < 0.001, **p < 0.01, and *p < 0.05. (**B**,**D**) Size distribution of spheres, whiskers show the minimal and maximal sphere size, the bottom and the top of the box show the firs and the third quartile, respectively. The line inside the box shows median size. The data are presented from one representative experiment.

SEM analyses indicated a progressive increase in sphere stability when a stronger ion concentration was used (Fig. 5). At the lowest ionic strength (0.5 M potassium phosphate), the sphere formation process was impaired. Particles were of undefined shape and formed aggregates. Their morphology became more spherical and they were more separated as the phosphate concentration increased. Additionally, the increasing phosphate concentration decreased the mean size of the particles (Fig. 6A) and narrowed their size distribution (Fig. 6B). These effects were clearly visible in the case of the PA-particles and observed as a slight trend in the case of the 80/20 spheres (Figs 5 and 6).

The size of the particles significantly depended on initial protein concentration (Figs 4 and 6C,D). As initial silk concentration changed from 0.5 mg/ml to 10 mg/ml, the mean size of produced spheres increased from 373 nm (±14 nm) to 1.19 μm (±193 nm) and 574 nm (±24 nm) to 1.94 μm (±0.84 μm) for MS2(9x) 80/20 and MS2(9x) PA silk, respectively (Fig. 6C). The differences in the mean sizes of spheres were significant for spheres prepared at the highest initial silk concentrations. Moreover, the highest initial concentrations of the silk produced spheres with a broad size distribution, what was more pronounced for MS2(9x) PA than MS2(9x) 80/20 spheres (Fig. 6D).

A 1.75 M potassium phosphate buffer adjusted to different pH values in the range of 6 to 12 did not influence the morphology or size of particles formed from MS2(9x) silk purified by either method (data not shown).

### Zeta potential (ZP)

**Figure 7:**
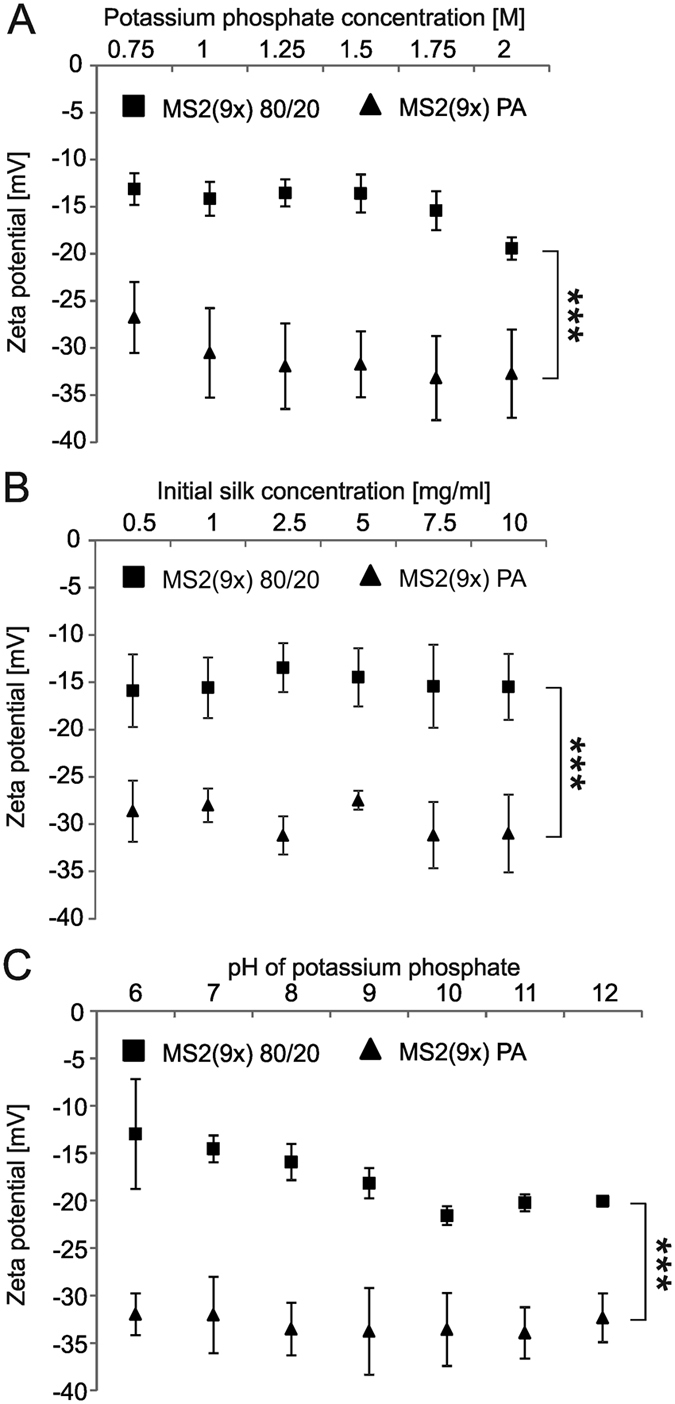
Zeta potential of the MS2(9x) 80/20 and MS2(9x) PA-spheres. (**A**) Spheres were prepared by mixing of: MS2(9x) silk protein (at initial concentration of 2.5 mg/ml) with various potassium phosphate concentrations, at pH 8 (**B**) silk protein at various initial concentrations with 2 M potassium phosphate, at pH 8; (**C**) silk protein (at 2.5 mg/ml) with 1.75 M potassium phosphate at various pH values. Zeta potential of silk spheres were measured three times in triplicate at 25 °C in 10 mM NaCl,***indicates statistical significance with p < 0.001.

Both MS2(9x) PA and MS2(9x) 80/20 spheres indicated a negative Zeta potential, which was in agreement with the calculated isoelectric point of the MS2(9x) protein (Ip: 5.27). Regardless of the particles’ production conditions (initial silk concentration, phosphate concentration and pH of phosphate buffer), a mean ZP of the MS2(9x) 80/20 spheres was approximately 2 times higher than one of MS2(9x) PA spheres (−15 and vs. −30 mV, respectively) (Fig. 7). In all processing conditions, the difference between the MS2(9x) 80/20 and MS2(9x) PA spheres was significant.

A slight drop in the ZP value was observed in the particles produced at higher potassium phosphate concentrations (Fig. 7A). The initial concentration of silk protein had no effect on the overall net charge of the sphere (Fig. 7B). The pH value of the phosphate buffer did not have a significant impact on the ZP of the MS2(9x) PA spheres. However, the zeta potentials of MS2(9x) 80/20 particles was changed towards more negative values, with pH values up to 10 (Fig. 7C).

### Secondary structure

**Figure 8:**
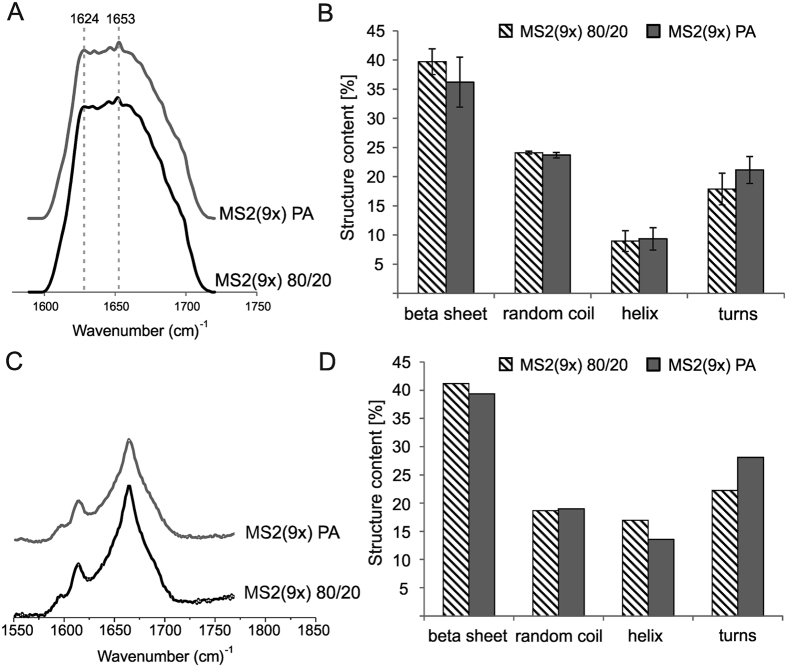
Secondary structure analysis of both variants of the MS2(9x) spheres. (**A**) FTIR and (**C**) Raman spectra of the MS2(9x) 80/20 and MS2(9x) PA spheres. (**A**) Bands at 1624 and 1653 cm^−1^ indicate β-sheet and random coil, respectively. (**B**,**D**) Secondary structure composition of the MS2(9x) 80/20 and MS2(9x) PA spheres calculated from FTIR (**B**) and Raman (**D**) spectra. Spheres were prepared by mixing 2.5 mg/ml with 2 M potassium phosphate at a pH of 8.

The secondary structure composition of the spider silk spheres was analyzed by FTIR and Raman spectroscopy (Fig. 8). The Amide I band of FTIR and Raman spectra showed a specific peak that indicated high crystallinity (β-sheet structure) in both samples. The calculated overall secondary structure content of both types of particles was very similar, whereas the β-sheet structure constituted approximately 40% (Fig. 8).

### Model drug loading

**Figure 9:**
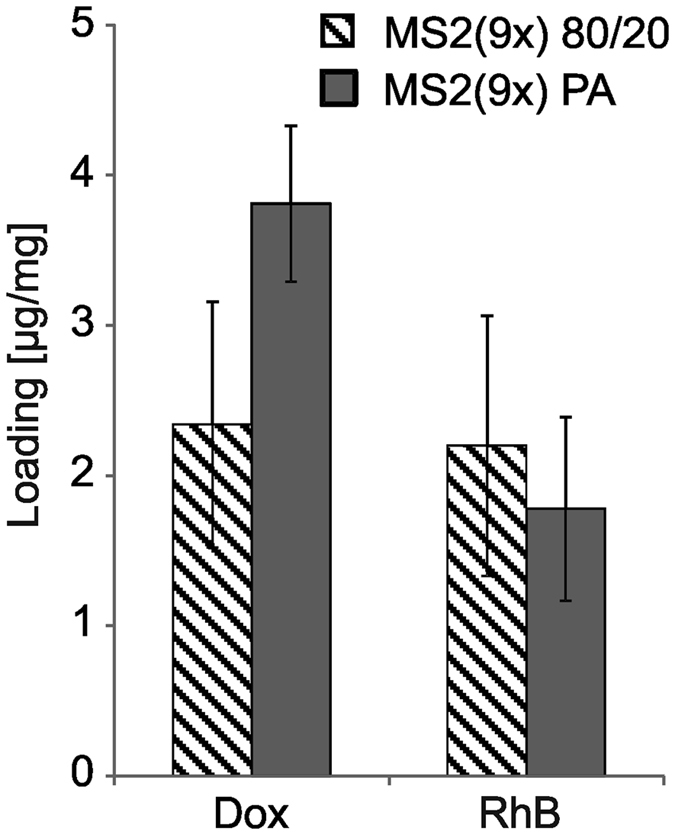
Loading of model drugs to the MS2(9x) 80/20 and MS2(9x) PA spheres. Spheres were prepared by mixing silk at an initial concentration of 10 mg/ml with 2 M potassium phosphate at a pH of 8. The amount of drugs loaded into 1 mg of spheres was calculated spectrophotometrically by measuring RhB and Dox absorbance at a wavelength of 554 nm and 508 nm, respectively. The experiment was repeated three times in triplicate.

A pilot study of model drug loading on the silk particles was performed. RhB was incorporated in similar amounts to both MS2(9x) 80/20 and MS2(9x) PA spheres. However, a cytotoxic anticancer drug, Dox, showed higher loading for the MS2(9x) PA silk spheres, with rates up to 3.9 μg per 1 mg of the spheres, compared with 2.3 μg/mg for MS2(9x) 80/20 particles (Fig. 9).

### Cytotoxicity

**Figure 10:**
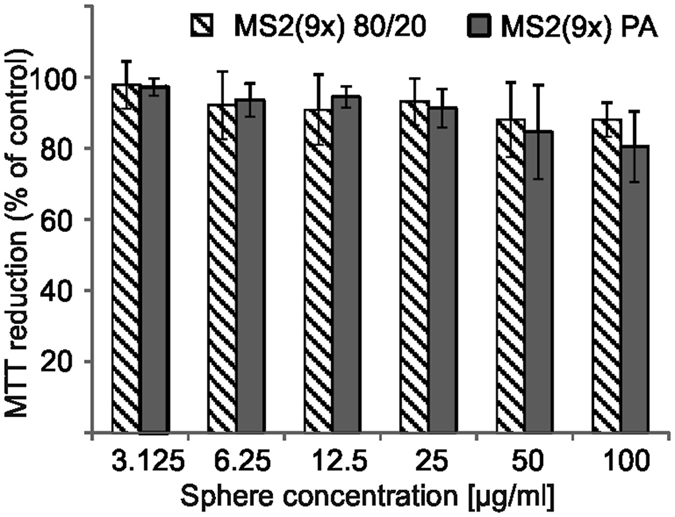
MS2(9x) sphere cytotoxicity towards NIH 3T3 fibroblasts studied by MTT assay. The percentage of the MTT reduction was referred to untreated cells (control). The error bars show the standard deviation of the mean from three independent experiments.

A cytotoxicity test was performed on the NIH 3T3 fibroblast cell line exposed to various sphere concentrations. After 48 h of incubation, neither type of silk spheres indicated significant reduction of the cells’ viability (Fig. 10).

## Discussion

In the present work, we designed a new bioengineered spider silk protein, MS2(9x), based on the MaSp2 spidroin of *N. clavipes* dragline silk. We studied the ability of these proteins to form spherical particles, with particular interest in the impact of the applied protein purification protocol on the particle properties. We purified MS2(9x) protein using two alternative methods: thermal and acidic extraction. Both methods produced silk proteins of similar quantity and purity but different in secondary structure and zeta potential value. The silk spheres made of these purified protein variants differed in such critical features as particle size, size distribution, morphology, ZP and drug binding potential.

Both protein variants: MS2(9x) 80/20 and MS2(9x) PA were composed of the same amino acid sequence and were purified from *E. coli* cells obtained from the same fermentor batch. MALDI-TOF spectra and SDS-PAGE electrophoresis of both variants of soluble silk protein showed that neither of them was truncated, multimerized, or contaminated with other proteins. However, FTIR, CD and Raman results indicated different secondary structure of the soluble MS2(9x) 80/20 and PA proteins. Moreover, the difference in the zeta potential of the soluble silk suggested that different amino acid residues might be exposed on the surface of both protein molecules, what also could be a result of different folding of these proteins. At this point it is not clear, whether difference in the composition of the secondary structure of both variants are due to the different physical/chemical processing conditions during purification process or due to any impurities that could be still present in the silk samples.

According to the CD spectra measured in the same protein concentration of 1 mg/ml, the content of the secondary structure of the MS2(9x) 80/20 and MS2(9x) PA proteins was differed. However, the obtained circular dichroism spectra have revealed that the secondary structure of both variants of the MS2(9x) proteins was dependent on the protein concentration. CD spectra of the lowest concentrations of studied proteins (0.125 mg/ml) had spectral characteristics of mostly disordered proteins, with very low ellipticity values above 210 nm and negative bands near 200 nm. The other bioengineered silk, eADF4(C16), purified by the thermal method also displayed a high content of random coil conformation when measured at concentration of 0.15 mg/ml^9^. A similar scheme of changes (shifts of minima) as observed in the circular dichroism spectra for MS2(9x) 80/20 and MS2(9x) PA proteins, was observed also in the case of oligomeric forms of human cystatin C (HCC)^29^,^30^. This protein forms toroidal oligomers (“donuts”) or fibrils probably through domain swapping mechanism, which had previously been described for dimers of native HCC^31^. Analysis of the secondary structure, conducted for this protein, showed a shift of the characteristic minimum in the CD spectra toward longer wavelengths (210–212 nm) both for HCC oligomers and fibrils. Thus, we can assume that observed for MS2(9x) 80/20 and MS2(9x) PA proteins differences in circular dichroism spectra may be related to the generation of a more compact and ordered structure of MS2(9x) PA.

Both studied bioengineered spider silks formed sphere-like structures when mixed with potassium phosphate at a concentration of 0.5 M, however these structures formed larger clusters of aggregates. In contrast, 0.75 M potassium phosphate triggered the formation of stable spheres. According to the mechanism proposed by Slotta *et al*. for eADF4(C16)^18^, the bioengineered silk proteins undergo a liquid-solid phase transition in presence of the phosphate ions. Upon mixing with phosphate buffer, the bioengineered silk proteins formed a protein-rich phase dispersed in the salt solution. The kosmotropic ions triggered the conformational change of the silk molecules and an increase in the intermolecular, hydrophobic interactions. The alanine-rich regions formed hydrogen bonds and induced the beta-sheet structure formation, that finally lead to the self-assembly of the proteins into spherical particles^18^. For both purified variants of MS2(9x) protein, the increasing potassium phosphate concentration caused the reduction of size and size distribution, however this effect was more pronounced for the MS2(9x) PA variant. Sphere formation upon mixing of silk solution with potassium phosphate was observed for several silks of different origin^15^,^18^,^19^,^22^. However, the concentration of potassium phosphate required to form stable silk spheres was different depending on silk variant (approximately 0.75 M for silkworm silk^15^, and 0.4 M for eADF4(C16)^18^). It can be a function of amino acid composition of silk, however it needs further examination–a direct comparison study is needed.

Ours and other studies indicated that the size of the spheres can be controlled by the initial concentration of the silk protein^15^,^19^. However, using equal initial concentrations of both proteins, the MS2(9x) PA spheres were always larger than the MS2(9x) 80/20 spheres. Moreover, spheres made of thermally purified silk (MS2(9x) 80/20) presented smooth surface and tightly packed, dense core. Their morphology resembled the most the morphology of previously reported eADF4(C16) bioengineered spider silk spheres^18^,^19^. Silk, purified using acidic method (MS2(9x) PA), formed porous spheres. The pores were visible on the sphere’s surface and penetrated the entire volume of the MS2(9x) PA spheres. Despite the differences in the secondary structure of soluble silk proteins (Fig. 2), both types of the silk spheres showed similar secondary structure composition (Fig. 8). According to the FTIR analysis, during the sphere formation process there was an approximately 14 and 3% increase in the β-sheet content for MS2(9x) 80/20 and PA spheres, respectively. The β-sheet component mainly increased at the expense of unordered structures and helices. This may suggest that during the process of forming a sphere, the structural changes were more pronounced for MS2(9x) 80/20 protein than for MS2(9x) PA variant. This might lead to better alignment of monomers of the MS2(9x) 80/20 protein and thus, promote tightly packed structure of the spherical particles. In case of the MS2(9x) PA protein, a part of beta-sheet structures has already been formed in the soluble form of the protein, so the protein monomers might have a limited capacity to closely align to other protein molecules. This might lead to formation of the porosity and bigger size of the MS2(9x) PA spheres.

The difference in zeta potential between the two types of spheres can result from the difference in their morphology. Independent on preparation conditions, the MS2(9x) PA spheres were always bigger and possessed pores, thus their overall surface was bigger than surface of the MS2(9x) 80/20 spheres. Another explanation may be an unequal amino acid residue distribution on the spheres surfaces. The ZP of MS2(9x) spheres presented in the Fig. 7C was measured in 10 mM NaCl, at the temperature of 25 °C. Spheres MS2(9x) 80/20 prepared at potassium phosphate of various pH values did not differed in size, however their ZP was lower for spheres formed at pH from 10 to 12 comparing with ZP of spheres formed at higher pH values. Thus, the difference in ZP was not due the measurement conditions or size of spheres. As mentioned above, during the sphere formation, the structural changes of the silk proteins took place, what finally might result in an unequal amino acid distribution on the spheres surfaces. Indeed, the structural changes were more pronounced for the MS2(9x) 80/20 than MS2(9x) PA spheres.

The pilot experiment on drug loading showed that the protein purification procedure eventually affected the drug loading efficiency. Rhodamine B was loaded with similar efficiency to both sphere types, which can be explain by its overall neutral charge^32^. The lower zeta potential of the MS2(9x) PA spheres may result in a twofold higher loading efficiency of the positively charged doxorubicin to the MS2(9x) PA spheres in comparison with the loading to MS2(9x) 80/20 spheres. Incorporation of doxorubicin may be explained by a diffusion process mediated by the electrostatic attraction as it was described for other silk particles^15^,^20^. However, we have previously shown, that the positively charged silk spheres were efficiently loaded with positive doxorubicin^22^. Therefore the mechanism of drug loading is probably not dependent exclusively on electrostatic attraction, but combines electrostatic and hydrophobic interactions.

The primary sequences of the natural major spidroins of *N. clavipes*: MaSp1 and MaSp2 share a common pattern; the central core is comprised of a large repetitive region flanked by short non-repetitive (NR) terminal domains. The NR domains control the protein solubility in the lumen and fiber formation process (reviewed by^33^,^34^). Moreover, the NR N-terminus contains the putative signal sequence for protein secretion. The micellar assemblies are most likely formed in the lumen, where NR domains are oriented towards the aqueous phase, whereas the repetitive regions are located inside the micelles to protect them from the solvent and from each other^33^,^34^. According to NMR studies on the natural spider silk isolated from the spider’s spinning gland, the highly repetitive core of the silk protein has a random and/or helical (polyproline-type II helix) conformation^35^,^36^. The MaSp1 and MaSp2 spidroins are stored in the lumen of the spinning gland at a ratio of 80:20^37^; however it was indicated that the conformation of both proteins is basically the same^36^. After secretion to the lumen of major ampullate glands, the natural spider silk proteins are stored at extremely high concentrations (up to 30–50% w%) without undesirable aggregation^35^. Next, spidroins are transported through the spinning duct where the fiber assembly process takes place. The assembly process involves a liquid-solid phase transition accompanied by structural changes of the spidroins. The final fiber contains crystalline regions (consist of β-sheet of polyalanine stretches) that are embedded in so-called amorphous matrix, which shows at least to moderate degree, a three-dimensional hierarchical order^38^. Because the MS2(9x) silk did not contain any signal sequence for secretion (either naturally or from an expression vector), it was accumulated in the bacterial cytoplasm. Absence of NR-termini in the bioengineered silk may be responsible for the lack of stabilization of the intrinsically disordered protein conformation. Thus, it is probable that during extensive overexpression in bacteria, silk may adopt the most thermodynamically stable conformation that also prevents undesirable aggregation. Moreover, the molecular chaperones of *E. coli*^39^,^40^ may assist the folding process of the bioengineered spider silk by binding to the hydrophobic, alanine rich domains. It may result in a particular structure of the MS2(9x) silk which may differ from the one observed in the lumen of a spinning gland. Next, the folding structure of the bioengineered spider silk protein can be altered during the purification process. Both purification protocols applied in the presented manuscript involved reagents and conditions that may potentially lead to disruption of the protein structure. The denaturing reagents were removed by dialyzing and during that process a new folding of silk protein could occur. Since the starting conditions of dialyze were different in both purification protocols, it might result in a different structure of the obtained proteins variants. Moreover, the purification process may also affect the properties of proteins because of the presence of the residual impurities. Although, the SDS-PAGE and mass-spectrometry results indicated, that the MS2(9x) silk variants were free of the bacterial proteins, other molecules such as nucleic acids and polysaccharides can be present in the protein solution. They may also interfere with the silk self-assembly process. Although, the key factor is not fully elucidated, eventually spheres produced from these purified variants differed in morphology and other properties. After additional purification process of soluble MS2(9x) 80/20 and MS2(9x) PA, both protein variants formed spheres of similar morphology. It indicated that the factor that influenced on the silk liquid-solid transition was eliminated.

## Conclusions

Independent of the purification method, neither type of MS2(9x) spheres were cytotoxic, which indicated that both methods can successfully be used for biomedical applications. However, this study highlights the impact that the employed purification method has on the structure of soluble silk, which may affect the aggregation and polymerization process and eventually determine the biomaterial’s properties. To obtain an artificial silk fiber that would possess the mechanical properties of natural one, it is critical to closely mimic native state of the proteins in the silk gland. The hierarchal silk transformation under specific salt composition, pH, dehydration and shear forces should result in proper polymerization into a fiber^34^. However, it is not the issue for other silk biomaterials including particles, capsules, hydrogels, films or scaffolds. The control of the purification process may be a new, attractive way to produce materials with novel characteristics.

## Methods

### Construction of the synthetic bioengineered silk gene–MS2(9x)

The MS2(9x) gene was designed based on the sequence encoding the consensus motif of the MaSp2 dragline silk of *N. clavipes*^41^. Complementary oligonucleotides encoding a monomer of the MS2(9x) gene MS2F (CTAGCGGTCCAGGCGGCTATGGTCCGGGCCAGCAAGGGCCGAGCGGTCCGGGCTCGGCGGCCGCGGCTGCGGCAGCGGCCGGACCTGGCGGCTATGGTCCGGGCCAGCAGA) and MS2R (CTAGTCTGCTGGCCCGGACCATAGCCGCCAGGTCCGGC-CGCTGCCGCAGCCGCGGCCGCCGAGCCCGGACCGCTCGGCCCTTGCTGGCCCGGACCATAGCCGCCTGGACCG) were synthesized (Integrated DNA Technologies, Leuven, Belgium). The *Nhe*I and *Spe*I restriction sites were included in the MS2 monomer for the multiple ligation cloning strategy. The oligonucleotides were annealed and then inserted into the *Nhe*I and *Spe*I restriction sites of the pETNX plasmid^10^. Next, the monomeric sequence was combined by using the multiple ligation technique utilizing *Spe*I inner restriction sites. The monomer was repeated 9 times in the final construct. The MS2(9x) construct was sequenced at the University of Adam Mickiewicz Core Facility, Poznan, Poland. Enzymes for digestion and ligation were supplied by Fermentas (St.Leon-Rot, Germany).

### Protein expression and purification

The plasmid carrying MS2(9x) construct (pETNX-MS2(9x)) was transformed into *E. coli* BLR (DE3) (Novagen, Madison, WI). For large scale expression, a Bioflo 3000 fermenter (New Brunswick Scientific, Edison, NJ) was used as described previously^10^. Two methods of purification, PA and 80/20, were applied to obtain MS2(9x) protein according to the previous study^10^.

#### 80/20 method (thermal method)

A bacterial pellet was suspended in a lysis buffer containing 20 mM HEPES (2-[4-(2-hydroxyethyl)piperazin-1-yl]ethanesulfonic acid) and 100 mM NaCl at a pH of 7.5 with a Protease Inhibitor Cocktail (2 mM AEBSF, 1 mM phosphoramidon, 130 mM bestatin, 14 mM E-64, 1 mM leupeptin, 0.2 mM aprotinin, and 10 mM pepstatin A) and 200 μg/ml lysozyme (Thermo Fisher Scientific, Inc., Waltham, MA) and was then incubated for 30 min on ice. Next, the lysate was sonicated using a Microson XL2000 sonicator (Misonics Inc. Farmingdale, NY) for 3 cycles of 10 s each. After supplementing the final concentrations with DNAse and MgCl_2_ with 100 μg/ml and 3 mM, respectively, it was incubated for 1 h on ice. Soluble bacterial proteins were precipitated by heat denaturation at 80 °C for 10 min and then were removed by centrifuging at 50 000 × g for 30 min at 4 °C. The supernatant was additionally denatured at 80 °C for 20 min, centrifuged, and after transfer to a clean vial, the ammonium sulfate was added to obtain a solution of 20% to precipitate the silk protein. After an overnight incubation at 4 °C with agitation, the silk protein was collected by centrifuging at 10 000 × g for 20 min at RT. The pellet was rinsed with 20% ammonium sulfate and was again centrifuged at 10 000 × g for 20 min at RT. The precipitated silk protein was dissolved in 6 M guanidine thiocyanate and then the silk solution was dialyzed against 10 mM Tris-HCl buffer, at a pH of 7.5 using a ZelluTrans Regenerated Cellulose Dialysis tube with the MWCO of 12–14 kDa (Carl Roth, Karlsruhe, Germany).

#### PA method (acid extraction method)

The bacterial pellet was treated with propionic acid. One milliliter of 13.3 M propionic acid was added per 1 g of the wet pellet, diluted to 2.3 M acid with ultrapure water and incubated with agitation for 1 h. To separate the acid-denatured bacterial proteins from the silk-containing solution, samples were centrifuged at 50 000 × g for 30 min at RT. Next, the supernatant was filtered and dialyzed against 10 mM Tris-HCl buffer with a pH of 7.5. Subsequently, the silk-containing solution was applied to a strong anion exchange Q-sepharose column (GE Healthcare Life Sciences Little Chalfont, UK) as described previously^10^.

Regardless of the type of silk purification method, the silk proteins were subsequently concentrated by ultrafiltration through a membrane with MWCO at 10 kDa (Millipore Centrifugal Filter Units, Millipore, Jaffrey, NH). The protein concentration was determined by UV spectroscopy referring to the extinction coefficient of 26820 cm^−1^ M^−1^. Quality of the purified protein was analyzed by separation in 12.5% SDS-PAGE gel with subsequent staining using Roti-Blue reagent (Carl Roth, Karlsruhe, Germany). The molecular weight was measured by MALDI-TOF spectrometry at the European Center of Bioinformatics and Genomics Core Facility, Poznan, Poland. Chemicals were supplied by Sigma (Sigma, St. Louis, MO) or as specified.

Additional processing of soluble silk proteins was applied when needed. A 20% ammonium sulfate was added to both solutions of MS2(9x) protein (5 mg/ml) purified with the thermal (80/20) and acid extraction (PA) methods to precipitate the previously purified silk. Next, the samples were centrifuged at 10 000 × g for 20 min at RT. The pellet was dissolved in 6 M guanidine thiocyanate and then dialyzed against 10 mM Tris-HCl buffer with a pH of 7.5 as described above.

### Preparation of silk spheres

The MS2(9x) spheres were formed by salting out with a potassium phosphate solution. One hundred microliters of MS2(9x) solution in 10 mM Tris with a pH of 7.5 was mixed with 1 ml of phosphate buffer using a pipette. Obtained spheres were incubated at RT for 2 h and then dialyzed overnight against ultrapure water or as indicated. For the sphere formation study, several processing conditions were applied: (i) variable initial concentration of silk: 0.5, 1, 2.5, 5, 10 mg/ml mixed with 2 M potassium phosphate with a pH of 8, (ii) variable concentration of phosphate: 0.5, 1, 1.25, 1.5, 1.75 and 2 M at a pH of 8.0 mixed with 2.5 mg/ml of MS2(9x), and (iii) 1.75 M phosphate solutions of different pH values: 6, 7, 8, 9, 10, 11, 12 mixed with 2.5 mg/ml of silk solution. Spheres for secondary structure analysis were obtained by mixing 2.5 mg/ml with 2 M potassium phosphate at a pH of 8. Spheres for FIB milling and model drug loading were prepared at an initial silk concentration of 10 mg/ml mixed with 2 M potassium phosphate at a pH of 8. The amount of spheres was determined gravimetrically.

### Scanning electron microscopy (SEM)

The spheres were air-dried on a glass slide and sputtered with gold/palladium for 60 s in a Quorum Sputter Coater Q150T ES (Quorum Technologies, Ringmer, UK). The spheres were analyzed under a JEOL JSM-7001F field emission scanning electron microscope (JEOL. Ltd, Tokyo, Japan) with 15 kV of accelerating voltage. At least 120 individual spheres were measured on 9 randomly selected SEM images to calculate an average particle size. The experiment was repeated at least three times.

### Focused ion beam (FIB)

The spheres were air-dried on a glass slide and sputtered with platinum for 45 s in a sputter coater Q150T ES (Quorum Technologies, Ringmer, UK). Focused ion beam milling was performed using a Ga^+^ ion beam at 30 kV, 23 pA and 17 nm in diameter. The ion beam was used to cross-section individual particles within the sample. After FIB milling, the specimens were transferred to the scanning electron microscope and imaged as specified above.

### Zeta potential measurement

The zeta potential was measured by a Zetasizer Nano XS (Malvern Instruments. Ltd, Worcestershire, UK). Soluble silk proteins were measured at the concentration of 2.5 mg/ml in 10 mM Tris-HCl at various pH values. The protein samples were loaded to the capillary cells using the diffusion barrier technique, according to the manufacturer’s instructions. Spheres for the zeta potential measurements were dialyzed against 10 mM NaCl. The samples were sonicated for 5 minutes directly before measurement in a sonic water bath. All measurements were performed three times in triplicate at 25 °C.

### FTIR spectroscopy

Two kinds of silk samples were examined: soluble (solution) and solid (spheres). The silk solution was centrifuged prior to analysis at 10 000 × g for 15 min. 50 μl of the silk protein solution (5 mg/ml in 10 mM Tris-HCl pH 7.5) was placed onto a triple reflection horizontal ATR attachment with a ZnSe crystal (Bruker Corp, Fremont, CA). For FTIR analysis, the silk spheres were lyophilized and blended with potassium bromide (KBr). The obtained powder was pressed under a pressure of 15 tones/cm^2^. Silk protein solutions and silk spheres in KBr pellets were analyzed by FTIR spectroscope Bruker TENSOR 27 (Bruker Corp, Fremont, CA) with a MCT detector. Absorption spectra were obtained with resolution of 2 cm^−1^ within a wavenumber range of 400 to 4000 cm^−1^. 512 scans were averaged. Background absorption of 10 mM Tris-HCl pH 7.5 buffer and pure KBr pellet was subtracted from the spectra of soluble silk protein and sphere-containing pellets, respectively. Second derivative calculation and deconvolution were performed with Opus 5.0 software (Bruker Corp. Fremont, CA). The second derivative was obtained from the amide I spectra region (range from 1595 to 1705 cm^−1^) by using a third degree polynomial function. Spectra analysis and curve fitting were performed using PeakFit Software (Systat Software, Inc. San Jose, CA). Fourier self-deconvolution of the amide I band was performed using the Lorenzian line slope and a half-brandwidth of 10.7 cm^−1^ and noise reduction factor of 0.45. The spectra were curve-fitted with a mixed Gaussian and Lorenzian peak shapes fit in positions determined by the 2^nd^ derivative spectrum minima. The secondary structure components were assigned to the amide I band components. The percentage of each secondary structure was assessed by summarizing the integrals of associated peaks and normalizing them to the total amide I integral. The experiment was repeated three times.

### Circular dichroism (CD) spectroscopy

The CD spectra of the MS2(9x) soluble proteins diluted in ultrapure water at concentrations from 0.125 to 1 mg/ml were acquired using a Jasco J-815 (Jasco Inc. Easton MD) circular dichroism spectropolarimeter in a 0.5 mm path length quartz high precision cell (Hellma Analytics, Müllheim, Germany). Spectra were recorded as an average of 10 scans in the range of 300–185 nm with a bandwidth of 0.5 nm at a scan rate of 50 nm/min. The background CD spectrum of ultrapure water was recorded and subtracted from sample spectra. The spectra were smoothed using a Savitzky-Golay filter with a convolution width of 9 points using Spectra Analysis software (Jasco Inc. Easton MD). The secondary structure of the soluble silk proteins were deduced from the CD spectra (expressed as mean residue ellipticity value) using the DICHROWEB online server^42^ and both CDSSTR and CONTIN analysis procedures^43^. The graphical presentation of deconvoluted CD spectra was performed using Origin package (Microcal Software, Inc., Northampton, MA, USA).

### Raman spectroscopy

Two kinds of silk samples were examined: soluble (solution) and solid (spheres). The nonpolarized Raman spectra were recorded in the back scattering geometry using the inVia Renishaw micro-Raman system (Reinshaw, Gloucestershire, UK). As an excitation of the incident light, the infrared solid state laser operating at 785 nm was used. The concentration of silk for analysis of soluble form was 5 mg/ml (in 10 mM Tris-HCl pH 7.5). To analyze silk spheres (5 mg/ml in Millipore water) with Raman spectroscopy, the laser beam was tightly focused on the silk sphere through a Leica × 50 long working distance (LWD) microscope objective with a numerical aperture of 0.5, leading to a laser beam diameter of approximately 2 μm (Leica Microsystems, Wetzlar, Germany). The position of the microscope objective with respect to the silk sphere sample was piezoelectrically controlled during the measurement (XY position). The reference position (level 0) was assumed for a laser spot focused on the surface of the sample. To prevent any damage of the sample an excitation power was fixed at 20 mW. The Raman spectra were recorded with a spatial resolution of approximately 2 μm. To increase the signal-to-noise ratio, the Raman spectra were accumulated. The average spectrum was calculated from at least 9 spectra recorded at different locations on the silk protein solution or sphere sample.

The Raman scattering spectra of the soluble silks and spheres were recorded within the spectral range of 1550–1750 cm^−1^. This frequency region corresponds to the Amide I Raman band position. Band spectral parameters related to secondary structure of the silk were obtained by deconvolution of the Amide I envelope in WIRE 3.1 (Renishaw) software. The Amide I Raman band was only used to assess the secondary structure of silk spheres because the Amide II and III Raman bands were too weak. From spectral decomposition of the Amide I Raman band, information about the structure of the silk, such as helix (1650 cm^−1^), β-sheet (1670 cm^−1^), turns (1685 cm^−1^) or unordered (1640 cm^−1^), was obtained^44^.

### Drug loading

One milligram of silk spheres was suspended in 1 ml of 25 μg/ml Rhodamine B (RhB) (Sigma, St. Louis, MO) solution in water or 25 μg/ml doxorubicin (Dox) (Adriamycin, Pfizer Inc., New York City, NY) in water and incubated overnight at RT with agitation at 300 RPM. Next, the spheres were centrifuged and the drug concentration in the supernatant was determined using UV-Vis spectrophotometry at λ = 554 nm or 508 nm for RhB and Dox, respectively. A standard calibration curve for the model drug was used for drug quantification. The total amount of the drug loaded into 1 mg of silk spheres was calculated by subtracting the amount of the drug remaining in the supernatant from the initially added amount. The experiment was repeated three times in triplicate.

### Cytotoxicity

A mouse cell line of NIH 3T3 fibroblasts were maintained in Dulbecco’s Modified Eagle Medium (Sigma, St. Louis, MO) supplemented with 10% fetal bovine serum (Sigma, St. Louis, MO) and 80 μg/ml gentamycin (KRKA Nove Mesto, Slovenia) in a humid atmosphere of 5% CO_2_ at 37 °C. The cells were seeded into 96-well plate in amount of 2.5 × 10^4^ cells/well (~78 cells/cm^2^) and were cultured for 24 h. MS2(9x) spheres were suspended in the culture media and added to the culture at final concentrations from 100 μg/ml to 3.1 μg/ml. The culture media without spheres was added to control wells. After 48 h of incubation, a MTT assay was performed. Briefly, 50 μl of 5 mg/ml (3-(4,5-dimethylthiazol-2-yl)-2,5-diphenyltetrazolium bromide (MTT) (BD Biosciences, San Jose, CA) was added to each well and incubated for 4 h. Next, the culture media was removed and 200 μl of DMSO was added to dissolve the purple formazan. After mixing, 100 μl of the solution was transferred to a new 96-well plate and the absorbance at λ = 570 nm was read on a microplate reader ELX808IV (Bio-Tek Instruments, Winooski, VT). The mitochondrial function and, by extension, the relative cell viability were presented as the percent of the negative control sample absorbance. The experiment was repeated three times (each n = 5).

### Statistics

The statistical significance of the differences between MS2 80/20 and MS2 PA groups was calculated using one way and two-way analysis of variance (ANOVA). In the case of significance (p < 0.05), post-hoc tests with Bonferroni correction were performed. The differences between groups were considered significant if the p value was below 0.05.

## Additional Information

**How to cite this article**: Jastrzebska, K. *et al*. The method of purifying bioengineered spider silk determines the silk sphere properties. *Sci. Rep.* **6**, 28106; doi: 10.1038/srep28106 (2016).

## Supplementary Material

Supplementary Information

## References

[b1] Kluge J. A., Rabotyagova O., Leisk G. G. & Kaplan D. L. Spider silks and their applications. Trends Biotechnol 26, 244–251 (2008). 18367277 10.1016/j.tibtech.2008.02.006

[b2] Tokareva O., Michalczechen-Lacerda V. A., Rech E. L. & Kaplan D. L. Recombinant DNA production of spider silk proteins. Microb Biotechnol 6, 651–663 (2013). 24119078 10.1111/1751-7915.12081 PMC3815454

[b3] Rising A., Widhe M., Johansson J. & Hedhammar M. Spider silk proteins: recent advances in recombinant production, structure-function relationships and biomedical applications. Cell Mol Life Sci 68, 169–184 (2011). 20668909 10.1007/s00018-010-0462-z PMC11114806

[b4] Scheibel T. Spider silks: recombinant synthesis, assembly, spinning, and engineering of synthetic proteins. Microb Cell Fact 3, 14 (2004). 15546497 10.1186/1475-2859-3-14 PMC534800

[b5] Bini E. . RGD-functionalized bioengineered spider dragline silk biomaterial. Biomacromolecules 7, 3139–3145 (2006). 17096543 10.1021/bm0607877

[b6] Hedhammar M. . Structural properties of recombinant nonrepetitive and repetitive parts of major ampullate spidroin 1 from Euprosthenops australis: implications for fiber formation. Biochemistry 47, 3407–3417 (2008). 18293938 10.1021/bi702432y

[b7] Rabotyagova O. S., Cebe P. & Kaplan D. L. Self-assembly of genetically engineered spider silk block copolymers. Biomacromolecules 10, 229–236 (2009). 19128057 10.1021/bm800930x

[b8] Jansson R. . Recombinant spider silk genetically functionalized with affinity domains. Biomacromolecules 15, 1696–1706 (2014). 24678858 10.1021/bm500114e

[b9] Huemmerich D. . Primary structure elements of spider dragline silks and their contribution to protein solubility. Biochemistry 43, 13604–13612 (2004). 15491167 10.1021/bi048983q

[b10] Dams-Kozlowska H. . Purification and cytotoxicity of tag-free bioengineered spider silk proteins. J Biomed Mater Res A 101, 456–464 (2013). 22865581 10.1002/jbm.a.34353 PMC3494781

[b11] Mello C. M., Soares J. W., Arcidiacono S. & Butler M. M. Acid extraction and purification of recombinant spider silk proteins. Biomacromolecules 5, 1849–1852 (2004). 15360297 10.1021/bm049815g

[b12] Lazaris A. . Spider silk fibers spun from soluble recombinant silk produced in mammalian cells. Science 295, 472–476 (2002). 11799236 10.1126/science.1065780

[b13] Spiess K., Lammel A. & Scheibel T. Recombinant spider silk proteins for applications in biomaterials. Macromol Biosci 10, 998–1007 (2010). 20602494 10.1002/mabi.201000071

[b14] Schacht K. & Scheibel T. Processing of recombinant spider silk proteins into tailor-made materials for biomaterials applications. Curr Opin Biotechnol 29, 62–69 (2014). 24657706 10.1016/j.copbio.2014.02.015

[b15] Lammel A. S., Hu X., Park S. H., Kaplan D. L. & Scheibel T. R. Controlling silk fibroin particle features for drug delivery. Biomaterials 31, 4583–4591 (2010). 20219241 10.1016/j.biomaterials.2010.02.024 PMC2846964

[b16] Seib F. P., Jones G. T., Rnjak-Kovacina J., Lin Y. & Kaplan D. L. pH-dependent anticancer drug release from silk nanoparticles. Adv Healthc Mater 2, 1606–1611 (2013). 23625825 10.1002/adhm.201300034 PMC3808531

[b17] Germershaus O., Werner V., Kutscher M. & Meinel L. Deciphering the mechanism of protein interaction with silk fibroin for drug delivery systems. Biomaterials 35, 3427–3434 (2014). 24461326 10.1016/j.biomaterials.2013.12.083

[b18] Slotta U. K., Rammensee S., Gorb S. & Scheibel T. An engineered spider silk protein forms microspheres. Angew Chem Int Ed Engl 47, 4592–4594 (2008). 18461576 10.1002/anie.200800683

[b19] Lammel A., Schwab M., Slotta U., Winter G. & Scheibel T. Processing conditions for the formation of spider silk microspheres. Chem Sus Chem 1, 413–416 (2008). 10.1002/cssc.200800030 18702135

[b20] Blüm C. & Scheibel T. Control of Drug Loading and Release Properties of Spider Silk Sub-Microparticles. Bionanoscience 2, 67–74 (2012).

[b21] Lammel A., Schwab M., Hofer M., Winter G. & Scheibel T. Recombinant spider silk particles as drug delivery vehicles. Biomaterials 32, 2233–2240 (2011). 21186052 10.1016/j.biomaterials.2010.11.060

[b22] Florczak A., Mackiewicz A. & Dams-Kozlowska H. Functionalized spider silk spheres as drug carriers for targeted cancer therapy. Biomacromolecules 15, 2971–2981 (2014). 24963985 10.1021/bm500591p

[b23] Kundu J., Chung Y. I., Kim Y. H., Tae G. & Kundu S. C. Silk fibroin nanoparticles for cellular uptake and control release. Int J Pharm 388, 242–250 (2010). 20060449 10.1016/j.ijpharm.2009.12.052

[b24] Chen M., Shao Z. & Chen X. Paclitaxel-loaded silk fibroin nanospheres. J Biomed Mater Res A 100, 203–210 (2012). 22021221 10.1002/jbm.a.33265

[b25] Wang X., Yucel T., Lu Q., Hu X. & Kaplan D. L. Silk nanospheres and microspheres from silk/pva blend films for drug delivery. Biomaterials 31, 1025–1035 (2010). 19945157 10.1016/j.biomaterials.2009.11.002 PMC2832579

[b26] Breslauer D. N., Muller S. J. & Lee L. P. Generation of monodisperse silk microspheres prepared with microfluidics. Biomacromolecules 11, 643–647 (2010). 20131893 10.1021/bm901209u

[b27] Wang X. . Silk microspheres for encapsulation and controlled release. J Control Release 117, 360–370 (2007). 17218036 10.1016/j.jconrel.2006.11.021

[b28] Rammensee S., Slotta U., Scheibel T. & Bausch A. R. Assembly mechanism of recombinant spider silk proteins. Proc Natl Acad Sci USA 105, 6590–6595 (2008). 18445655 10.1073/pnas.0709246105 PMC2373321

[b29] Wahlbom M. . Fibrillogenic oligomers of human cystatin C are formed by propagated domain swapping. J Biol Chem 282, 18318–18326 (2007). 17470433 10.1074/jbc.M611368200

[b30] Ostner G. . Stabilization, characterization, and selective removal of cystatin C amyloid oligomers. J Biol Chem 288, 16438–16450 (2013). 23629649 10.1074/jbc.M113.469593 PMC3675580

[b31] Janowski R. . Human cystatin C, an amyloidogenic protein, dimerizes through three-dimensional domain swapping. Nat Struct Biol 8, 316–320 (2001). 11276250 10.1038/86188

[b32] Milanova D., Chambers R. D., Bahga S. S. & Santiago J. G. Electrophoretic mobility measurements of fluorescent dyes using on-chip capillary electrophoresis. Electrophoresis 32, 3286–3294 (2011). 22102501 10.1002/elps.201100210

[b33] Hagn F. A structural view on spider silk proteins and their role in fiber assembly. J Pept Sci 18, 357–365 (2012). 22570231 10.1002/psc.2417

[b34] Rising A. Controlled assembly: a prerequisite for the use of recombinant spider silk in regenerative medicine? Acta Biomater 10, 1627–1631 (2014). 24090990 10.1016/j.actbio.2013.09.030

[b35] Hijirida D. H. . 13C NMR of Nephila clavipes major ampullate silk gland. Biophys J 71, 3442–3447 (1996). 8968613 10.1016/S0006-3495(96)79539-5 PMC1233831

[b36] Lefevre T. . *In situ* conformation of spider silk proteins in the intact major ampullate gland and in solution. Biomacromolecules 8, 2342–2344 (2007). 17658884 10.1021/bm7005517

[b37] Brooks A. E., Steinkraus H. B., Nelson S. R. & Lewis R. V. An investigation of the divergence of major ampullate silk fibers from Nephila clavipes and Argiope aurantia. Biomacromolecules 6, 3095–3099 (2005). 16283732 10.1021/bm050421e

[b38] Heim M., Romer L. & Scheibel T. Hierarchical structures made of proteins. The complex architecture of spider webs and their constituent silk proteins. Chem Soc Rev 39, 156–164 (2010). 20023846 10.1039/b813273a

[b39] Gatenby A. A., Viitanen P. V. & Lorimer G. H. Chaperonin assisted polypeptide folding and assembly: implications for the production of functional proteins in bacteria. Trends Biotechnol 8, 354–358 (1990). 1369447 10.1016/0167-7799(90)90224-l

[b40] Baneyx F. & Mujacic M. Recombinant protein folding and misfolding in Escherichia coli. Nat Biotechnol 22, 1399–1408 (2004). 15529165 10.1038/nbt1029

[b41] Hinman M. B. & Lewis R. V. Isolation of a clone encoding a second dragline silk fibroin. Nephila clavipes dragline silk is a two-protein fiber. J Biol Chem 267, 19320–19324 (1992). 1527052

[b42] Whitmore L. & Wallace B. A. Protein secondary structure analyses from circular dichroism spectroscopy: methods and reference databases. Biopolymers 89, 392–400 (2008). 17896349 10.1002/bip.20853

[b43] Sreerama N., Venyaminov S. Y. & Woody R. W. Estimation of protein secondary structure from circular dichroism spectra: inclusion of denatured proteins with native proteins in the analysis. Anal Biochem 287, 243–251 (2000). 11112270 10.1006/abio.2000.4879

[b44] Lefevre T., Rousseau M. E. & Pezolet M. Protein secondary structure and orientation in silk as revealed by Raman spectromicroscopy. Biophys J 92, 2885–2895 (2007). 17277183 10.1529/biophysj.106.100339 PMC1831708

