# Supplementary material for: The method of purifying bioengineered spider silk determines the silk sphere properties
Source: Sci Rep. 2016 Jun 17;6:28106. doi: 10.1038/srep28106 (PMC4911573; doi:10.1038/srep28106)
Supplement: Supplementary Information [file srep28106-s1.pdf]

The method of purifying bioengineered spider silk determines the silk sphere properties

Katarzyna Jastrzebska[1,2], Edyta Felcyn[3], Maciej Kozak[4,5], Miroslaw Szybowicz[6], Tomasz Buchwald[6], Zuzanna Pietralik[4], Teofil Jesionowski[7], Andrzej Mackiewicz[1,3,8], Hanna Dams-Kozlowska[1,8, *]

[1] Chair of Medical Biotechnology, Poznan University of Medical Sciences, 61-688 Poznan, Poland
[2] NanoBioMedical Centre, Adam Mickiewicz University, 61-614 Poznan, Poland
[3] BioContract Sp. z o.o., 61-051 Poznan, Poland
[4] Department of Macromolecular Physics, Adam Mickiewicz University, 61-614 Poznan, Poland
[5] Joint Laboratory for SAXS Studies, Adam Mickiewicz University, 61–614 Poznan, Poland
[6] Faculty of Technical Physics, Poznan University of Technology, 60-965 Poznan, Poland
[7] Faculty of Chemical Technology, Institute of Chemical Technology and Engineering, Poznan University of Technology, 60-965 Poznan, Poland
[8] Department of Diagnostics and Cancer Immunology, Greater Poland Cancer Centre, 61-688 Poznan, Poland

SUPPLEMENTARY INFORMATION

CD spectra recorded for the both soluble spider silk proteins at concentration of 1 mg/ml were deconvoluted using DICHROWEB server and CDSSTR and CONTIN analysis programs. Each CD data set was independently deconvoluted at least five times, using two reference data sets optimised for the recorded spectral range, i.e. data set 3 and set 6 (185-240 nm). Altogether we obtained around 20 sets of results of calculated secondary structure fractions, which were used to get proper statistics and standard deviation values. The exemplary extended results, containing the reconstructed data and normalized root mean square deviation (NRMSD), are presented in Table S1 and Fig. S1 for MS2(9x) PA protein and Table S2 and Fig. S2 for MS2(9x) 80/20 protein. The reconstructed CD data calculated on the basis of CDSSTR program were characterised by the lowest NRMSD values and the results obtained on its basis were prevailing in the calculation of the average secondary structure content.

MS2(9x) PA - concentration 1 mg/ml

Supplementary Table S1. The exemplary deconvolution data for MS2(9x) PA protein at concentration of 1 mg/ml obtained with CDSSTR and CONTIN programs and two reference data sets.

	MS2(9x) PA - 1 mg/ml							
	NRMSD	helix 1	helix 2	strand 1	strand 2	turns	random	total
CDSSTR/set 3	0.112	0.03	0.06	0.28	0.13	0.21	0.29	1
CDSSTR/set 6	0.074	0.02	0.06	0.24	0.11	0.17	0.41	1
CONTIN/set 3	0.213	0.05	0.044	0.28	0.136	0.213	0.277	1
CONTIN/set 6	0.216	0.04	0.031	0.242	0.121	0.151	0.415	1

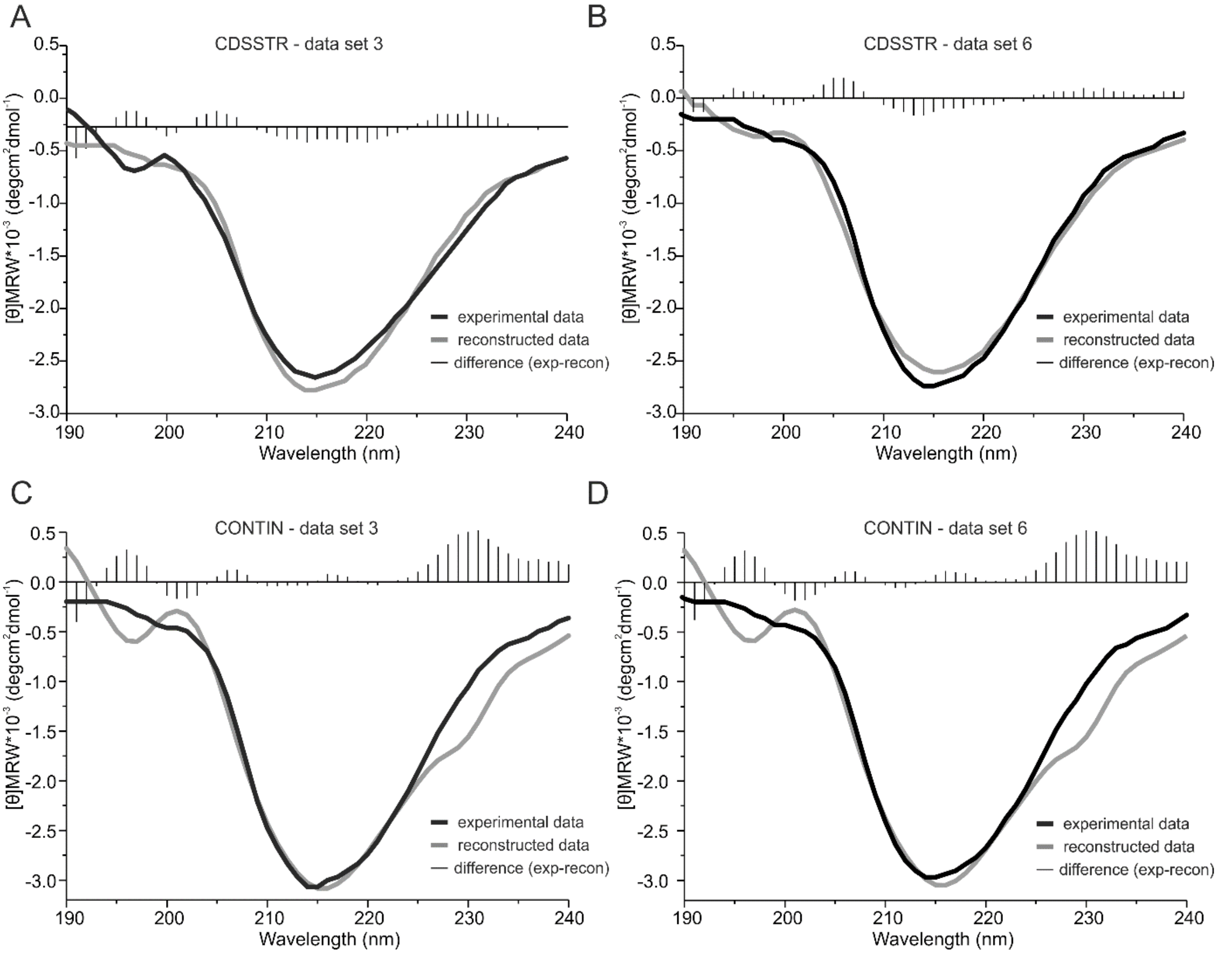

Supplementary Figure S1. Experimental and reconstructed circular dichroism data with the superimposed difference between these signals for MS2(9x) PA protein. The results of spectra deconvolution obtained by the use of CDSSTR analysis program with reference data set 3 (A)

or set 6 (B) and results obtained by the use of CONTIN analysis program with reference data set 3(C) and 6 (D) respectively. Deconvolution results are summarised in the Table S1**.**

MS2(9x) 80/20 - concentration 1 mg/ml

Supplementary Table S2. The exemplary deconvolution data for MS2(9x) 80/20 protein at concentration of 1 mg/ml obtained with CDSSTR and CONTIN programs and two reference data sets.

	MS2(9x) 80/20 - 1 mg/ml							
	NRMSD	helix 1	helix 2	strand 1	strand 2	turns	unordered	total
CDSSTR/set 3	0.13	0.03	0.03	0.26	0.13	0.22	0.34	1
CDSSTR/set 6	0.081	0.02	0.03	0.24	0.10	0.17	0.44	1
CONTIN/set 3	0.202	0.04	0.043	0.251	0.113	0.18	0.373	1
CONTIN/set 6	0.2	0.008	0.049	0.218	0.105	0.174	0.446	1

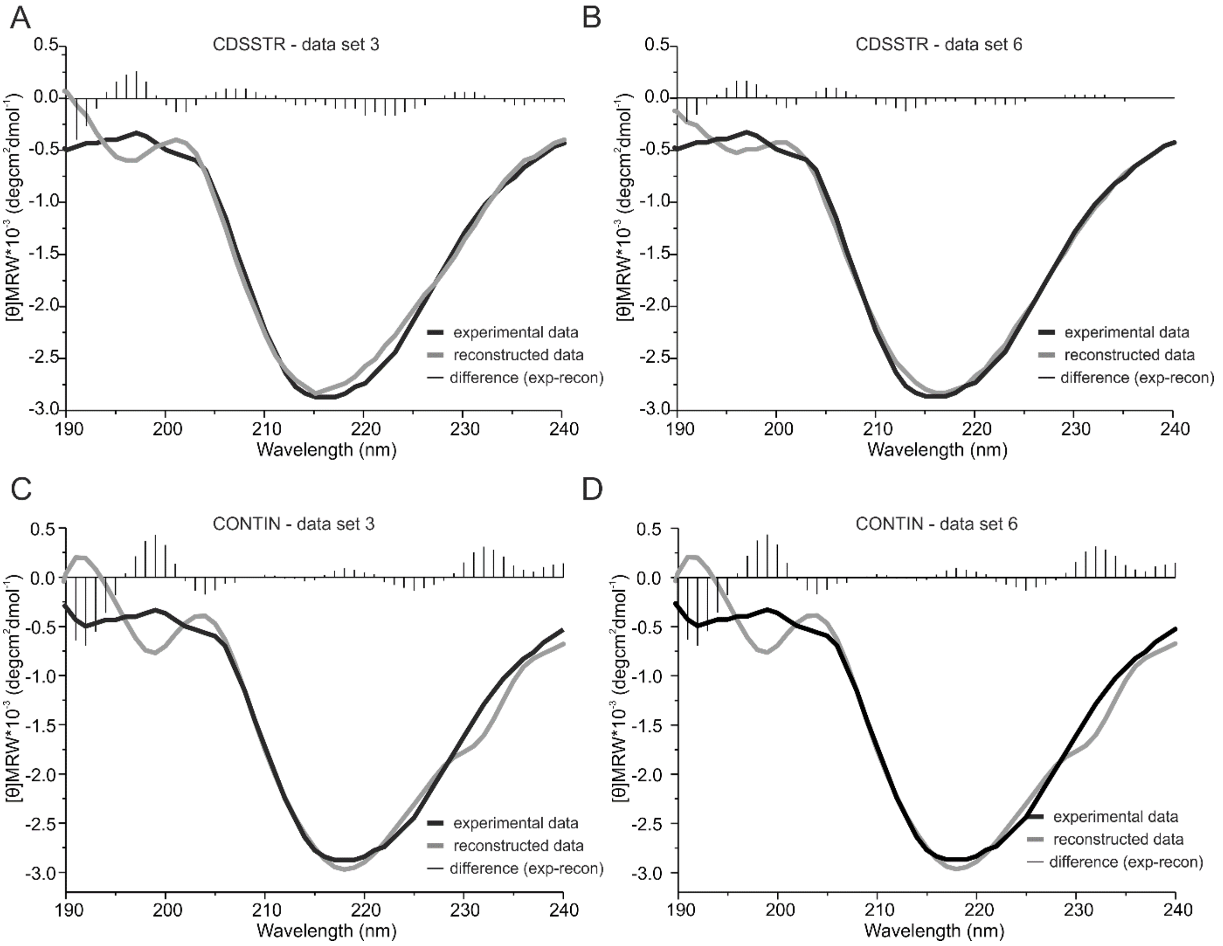

Supplementary Figure S2. Experimental and reconstructed circular dichroism data with the superimposed difference between these signals for MS2(9x) 80/20 protein. The results of spectra deconvolution obtained by the use of CDSSTR analysis program with reference data

set 3 (A) or set 6 (B) and results obtained by the use of CONTIN analysis program with reference data set 3(C) and 6 (D) respectively. Deconvolution results are summarised in the Table S2
